# Neglected Rupture of the Patellar Tendon After Fixation of Tibial Tubercle Avulsion in an Adolescent Male Managed With Ipsilateral Semitendinosus Autograft Reconstruction

**DOI:** 10.7759/cureus.15368

**Published:** 2021-06-01

**Authors:** Andreas Panagopoulos, Panagiotis Antzoulas, Savvas Giakoumakis, Anna Konstantopoulou, George Tagaris

**Affiliations:** 1 Orthopaedic Department, University of Patras, Patras, GRC; 2 Orthopaedic Department, Karamandeio Children’s Hospital, Patras, GRC

**Keywords:** avulsion, patellar tendon, neglected, tibial tubercle fracture, semitendinosus tendon, reconstruction

## Abstract

The combination of a tibial tubercle fracture with patellar tendon avulsion in adolescents is an extremely rare injury that needs to be managed properly. Herein, we report the case of a 15-year-old boy who presented to our department two months after sustaining a tibial tubercle fracture that had been managed with mini-open reduction and internal fixation in another hospital; he had restricted range of motion and complete inability to extend his knee. Clinical and radiological investigations revealed a neglected avulsion of the patellar tendon with marked scarring and severe retraction. The patient underwent patellar tendon reconstruction using the ipsilateral semitendinosus tendon that passed through separate tunnels in the patella and proximal tibia. The postoperative course was uneventful, and one year later the patient had a satisfactory range of motion and a Lysholm score of 90. To our knowledge, a neglected patellar tendon avulsion after tibial tubercle fracture fixation has been reported only once in the literature. The reconstruction of the patellar tendon using an ipsilateral semitendinosus autograft is an excellent surgical technique, especially when severe tendon retraction has occurred.

## Introduction

Avulsion fractures of the tibial tubercle are unusual but well-recognized injuries, representing less than 1% of all physeal fractures [1]. They are more common in male adolescents between 13 and 16 years of age, typically occurring after a violent eccentric contraction of the quadriceps muscle during jumping or landing; they may also occur spontaneously during running in more obese patients [1,2]. Sometimes, they are associated with Osgood-Schlatter disease and can present with a marked displacement of the apophysis, comminution, intra-articular extension, meniscal injuries, compartment syndrome, and avulsion of the patellar tendon [1,3].

Rupture of the patellar tendon is another uncommon injury in the pediatric population, with an estimated incidence of 7% of all extensor mechanism injuries [4]. As the physis is the weakest link of the muscle-tendon-bone interface during childhood, a tibial tubercle avulsion is much more likely to occur than a patellar tendon rupture. The combination of a tibial tubercle fracture with patellar tendon avulsion is even rarer, with only a few cases reported in the literature [5-14]. The tibial tuberosity fracture was first classified by Watson-Jones [15] before being modified into A and B subtypes by Ogden et al. in 1980 [16]. The first documented description of a combined tibial tubercle fracture with patellar tendon avulsion was reported in 1982 by Mayba [5], while Frankl et al. [8] later added a subtype C for describing simultaneous avulsion of the patellar tendon.

Herein, we present a case of a neglected patellar tendon rupture in an adolescent patient after fixation of a tibial tubercle avulsion, which was managed with semitendinosus autograft reconstruction. To the best of our knowledge, only one similar case has been reported in the literature. The authors obtained the patient’s and parents’ informed written consent for print and electronic publication of the case report.

## Case presentation

**Figure 1:**
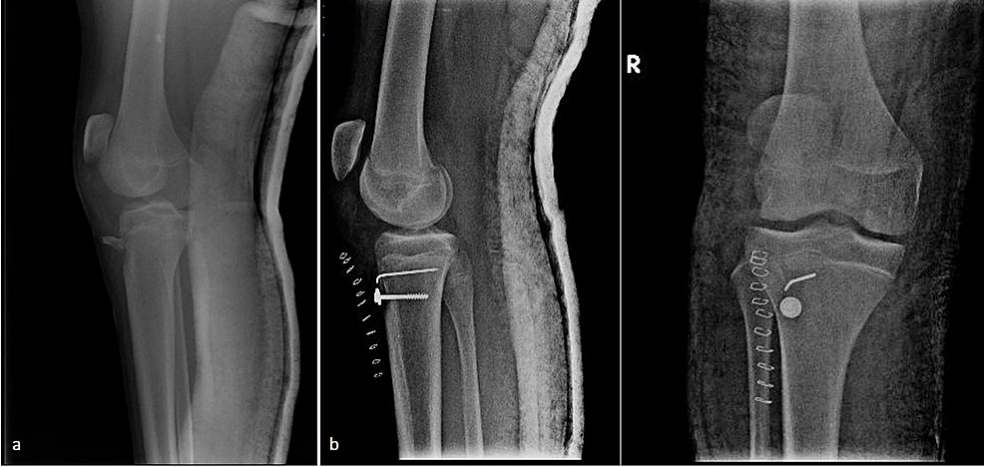
Initial radiographs. (a) Preoperative lateral radiography of the patient’s right knee showing a displaced tibial tubercle fracture (Ogden type IB); (b) postoperative lateral and anteroposterior radiographs after open reduction and internal fixation of the tuberosity with a cortical screw and one additional KW. This fixation was performed in another hospital and we did not have any information regarding the status of the patellar tendon at that time. KW: Kirschner wire

**Figure 2:**
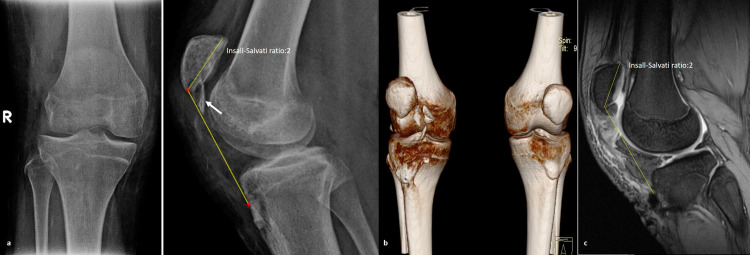
Radiological workup. (a) Anteroposterior and lateral radiographs showing a healed tuberosity fracture with evidence of patella alta (with the knee in 30° of flexion) and ossification of the patella tendon probably due to the previous operation (white arrow); (b) 3D-CT scan of both knees showing a high-riding patella on the right and multiple bone fragments or ossified parts of the tendon; (c) MRI scan of the affected knee demonstrating avulsion of the patella tendon with severe scarring and retraction and obvious patella alta. 3D-CT: three-dimensional-computed tomography

**Figure 3:**
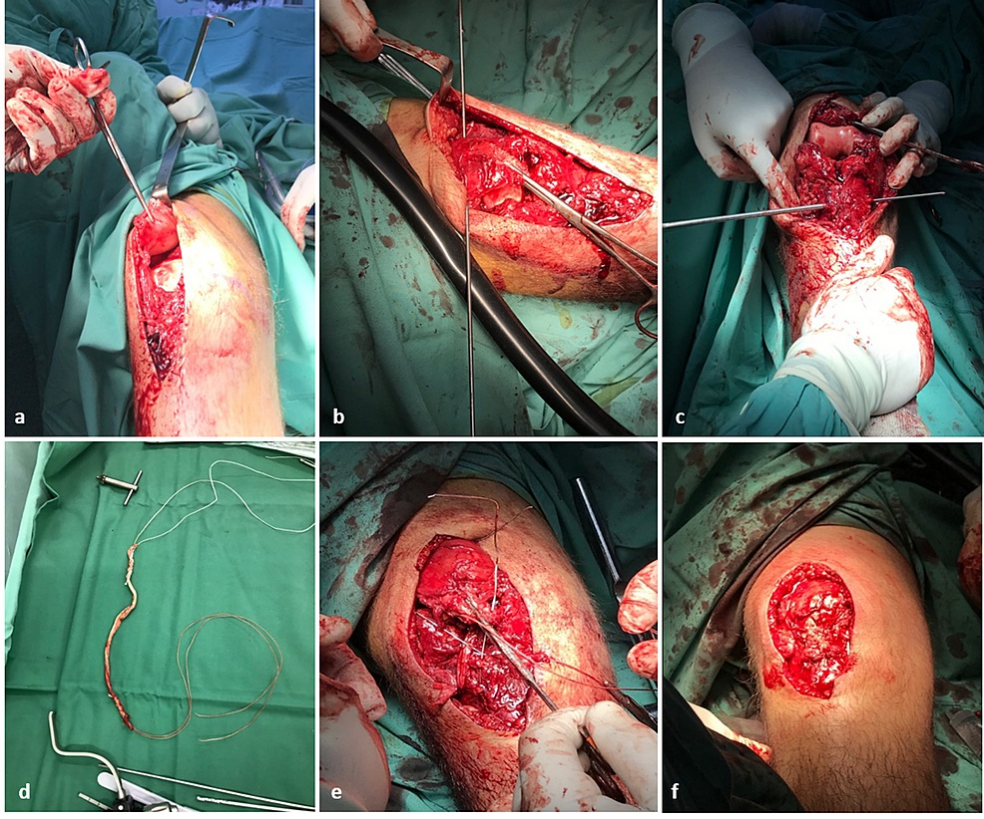
Intraoperative images. Intraoperative images of the applied surgical technique. (a) After an extensive open approach, the patella tendon was found scarred and retracted; (b, c) preparation of bone tunnels at the patella and below tuberosity in respect; (d) harvesting and preparation of the semitendinosus tendon; (e) passage of the graft through bone tunnels in a figure of eight fashion and additional reinforcement with a cerclage wire; (f) final construct prior to closure with retinaculum repair.

**Figure 4:**
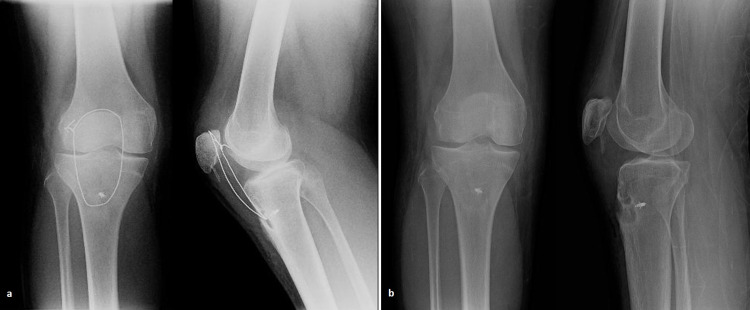
Postoperative and late follow-up radiographs. (a) Postoperative anteroposterior and lateral radiographs at one-week follow-up showing correction of patella alta; (b) final follow-up radiographs at one year showing maintenance of the reduction.

A 15-year-old adolescent with no significant medical or surgical history presented to the outpatient office with limping and inability to extend his right knee. He experienced a fall while cycling two months earlier and had been managed elsewhere with a mini-open reduction and internal fixation for an Ogden’s type IB fracture of the tibial tubercle using a 3.5-mm cannulated screw and one additional Kirschner wire; he was immobilized in a full extension plaster cast for five weeks, after which he was allowed partial weight-bearing. The patient underwent hardware removal on the seventh postoperative week in the treating hospital (Figures 1a, 1b). On physical examination, there was minimal soft tissue swelling around the knee, atrophy of the quadriceps, a palpable infrapatellar gap, a positive “camelback” sign at 30° of flexion, and an asymmetrically high-riding patella on palpation compared to the contralateral extremity. He was also unable to raise the leg straight. There were no signs of neurovascular compromise or inflammation. Subsequently, radiological examination revealed a healed tuberosity fracture and an obvious patella alta with an Insall-Salvati ratio of 2 (Figure 2a). No evidence of Osgood-Schlatter disease was found, but an ossification of the patellar tendon near the distal patellar pole was noted. Complete avulsion of the patellar tendon, along with severe retraction and scarring formation, was confirmed with three-dimensional-computed tomography (3D-CT) and magnetic resonance imaging (MRI) (Figures 2b, 2c). After obtaining parental informed consent, the patient was transferred the following day to the operative theater for patellar tendon reconstruction using the ipsilateral semitendinosus tendon. The patient was placed in a supine position with a high thigh tourniquet and was given general anesthesia. After proper limb preparation and draping, a second dose of cephalosporin was administered for infection prophylaxis, the limb was exsanguinated, and the tourniquet was inflated. A longitudinal midline incision was made over the anterior aspect of the knee, extending from the upper pole of the patella to 3 cm below the tibial tubercle. The patellar tendon was scarred at its entire length, ossified at its proximal part, and retracted, with less than 4 cm of viable proximal stump remaining, measured with a ruler (Figure 3a). The anterior cruciate ligament (ACL) guide wire (2.7 mm) and a cannulated ACL drill (7 mm diameter) were used for bone tunnel preparation both at the midline of the patella as well as 2 cm below the tibial tubercle (Figures 3b, 3c). A semitendinosus tendon autograft, measuring 6 mm in diameter, was harvested from the ipsilateral knee according to the standard ACL preparation; it was whipstitched at both ends with special loop sutures (Figure 3d). The tendon autograft, after proper irrigation in hydrogen peroxide for 1 minute and soaking in 500 mg vancomycin solution, was passed through both tunnels in a figure-eight fashion. After applying the appropriate tension, the patella alta was corrected, allowing the knee to flex to 60° without difficulty or undue tension on the repaired tendon. The graft was sutured to itself at 20° of flexion and the fixation point was augmented with a Mitek bone anchor (Figure 3e). The remnants of the patellar tendon were incorporated into the graft. An additional cerclage wire was passed through the tibial tunnel and at the insertion of the quadriceps tendon in the proximal patella pole for further reinforcement of the reconstruction. The wound was irrigated, the tourniquet was deflated, hemostasis was achieved, and a multilayered closure was performed. The remaining retinaculum was repaired using absorbable sutures (Figure 3f). Postoperative radiographs showed correction of the patella alta (Figure 4a). The knee was immobilized in full extension for two weeks using a hinged knee brace, and partial weight-bearing was tolerated with the support of crutches. After two weeks, the patient was encouraged to perform passive-assisted flexion up to 60° but was kept on crutches for full weight-bearing. Every two weeks, flexion was increased by 20-30°. The brace was removed after 12 weeks, and the cerclage wire was removed on the 16th postoperative week. One year postoperatively, the patient had a satisfactory range of motion (0° to 140°) and a Lysholm score of 90. The final radiological evaluation revealed that the patella was in the proper position (Figure 4b).

## Discussion

Simultaneous occurrence of tibial tubercle fracture and patellar tendon avulsion is extremely rare. Ali Yousef and Rosenfeld [4] reported an incidence of 4% (three cases) among 71 patients with traumatic injuries to their extensor mechanism. In contrast, Bauer et al. [17] reported five cases (23%) in their review of 22 patients with tibial avulsion fractures. This injury mainly affects adolescents (aged 13 to 16 years), which is consistent with the time of secondary ossification of the tibial tubercle apophysis; there is only a single report in the literature describing tibial tuberosity and patellar tendon avulsion in an adult patient [18].

Most reported injuries were associated with sports activities during basketball [6,8,12,14], football or soccer [10,13], skateboarding [11], running [5,14], and jumping [7,9,12]. The two traction apophysitis diseases of the knee, Osgood-Schlatter disease and Sinding-Larsen-Johansson syndrome, are postulated to be associated with avulsion fractures of the tibial tubercle [10,12]. According to the first description reported by Mayba [5], the mechanism of injury is a violent continued contraction of the quadriceps muscle even after a tuberosity fracture has occurred. Frankl et al. [8] considered that a violent flexion of the knee against a tightly contracted quadriceps muscle, when the foot is in a fixed position, is the main cause of this type of injury; another possible mechanism is the violent contraction of the quadriceps during extension, such as when a person is jumping [10]. Tibial tuberosity fractures are classified into three types according to Watson-Jones [15] and the later modification of a study by Ogden et al. [16]: type I fractures across the secondary ossification center at the level with the posterior border of the inserting patellar ligament, type II fractures at the junction of the primary and secondary ossification centers of the proximal tibial epiphysis, and type III fractures that propagate across the primary ossification center of the proximal tibial epiphysis into the knee joint. Each type is divided into two subtypes, A and B, depending on the severity of displacement and comminution. Frankl et al. [8] suggested a type C classification to the modified Ogden system for concurrent tibial tuberosity fracture and patellar tendon avulsion, as is the case in the patient presented here.

The presence of a patellar tendon avulsion has significant management implications, and clinical diagnosis may be challenging in acute cases due to increased pain and diffuse swelling. An asymmetrically high-riding patella on palpation compared to the contralateral extremity along with the inability of the patient to extend the knee against gravity are the most important clinical tests [10,12,13]. No such information was available in this case because the patient was initially treated elsewhere. It seems unlikely that the patella tendon rupture was caused during the fixation of the tibial tubercle avulsion. The initial lateral X-ray showed a high-riding patella that remained the same after tubercle fixation. Lateral knee radiography can easily detect tuberosity fractures, but a high level of suspicion is recommended to accurately diagnose synchronous patella tendon avulsion; patella alta (Insall-Salvati ratio >1.2) at 30° of knee flexion and multiple fragments at the distal patella pole are critical radiological signs that can establish the diagnosis. Both signs were present in our patient two months after initial management. In uncertain cases, the use of an MRI scan has been proposed to not only confirm the patellar tendon avulsion but also to investigate concomitant knee pathologies such as meniscal, cartilage, collateral ligament injuries, or even acute compartment syndrome [10,19]. A postoperative MRI scan is also useful to investigate tendon remodeling and proper attachment of the graft, but our patient and his parents refused to undergo the examination.

The management of acute bifocal tibial tuberosity injuries (type C) is surgical, aiming to anatomically reduce the tuberosity fragment and restore the disrupted extensor mechanism of the knee. The tibial tuberosity fragment can be fixed with screws [6-14], staples [12], or transosseous sutures [5], whereas the patella tendon can be repaired with standard Krakow sutures [5,6,8,10-13], staples [7,9], or bone anchors [14]. Reinforcement of ligament repair has been proposed by some authors to be done using either a cerclage wire [10] or a fiber wire through a patella bone tunnel [11]. Pereira et al. [6] performed reinforcement of the repair in a 15-year-old basketball player using an autologous semitendinosus autograft fixed distally with bone anchors and one screw. In cases of neglected patellar tendon injuries, reconstruction can be performed using the contralateral patellar tendon, turndown quadriceps graft, hamstring graft with preserved distal insertion, Achilles tendon allograft, or synthetic ligament augmentation [20]. Takazawa et al. [20] reported two cases of neglected patellar tendon ruptures: one in a 43-year-old male and the other in a 16-year-old boy who had been managed with open reduction and cannulated screw fixation for a tibial tuberosity avulsion at another institution 10 months prior. Similar to these cases, the patient discussed here presented with evidence of quadriceps wasting and a palpable gap below the patella. Subsequent plain radiography, 3D-CT, and MRI confirmed the presence of a severe patella alta (Insall-Salvati index, 1.6), massive ectopic calcification along the patellar tendon, and marked scarring and retraction. The semitendinosus and gracilis tendons, while preserved at their distal insertion, were used to reconstruct the patellar tendon, and the free end of the semitendinosus tendon was passed through a tunnel in the tibial tubercle (medial to lateral). The free ends of both tendons were then brought up proximally, crossing in front of the patella in a figure of eight fashion, before both were passed transversely through the distal end of the quadriceps tendon and pulled distally. Finally, both tendons were treated with interrupted sutures where they overlapped. To our knowledge, this is the only case in the literature reporting a neglected patellar tendon avulsion after operative fixation of a tibial tubercle fracture.

## Conclusions

Simultaneous occurrence of tibial tubercle fracture and patellar tendon avulsion occurs in a very low percentage of adolescents with a traumatic injury of the knee extensor mechanism. Strong clinical suspicion of patellar tendon avulsion and/or radiological evidence of patella alta in the preoperative workup requires either an additional MRI investigation or a routine extensile approach for tuberosity fixation, which allows for proper inspection and subsequent repair of the patella tendon avulsion. In neglected cases, ipsilateral semitendinosus autograft reconstruction can lead to a successful final outcome.
